# Relevance of multilamellar and multicompartmental vesicles in biological fluids: understanding the significance of proportional variations and disease correlation

**DOI:** 10.1186/s40364-023-00518-0

**Published:** 2023-08-26

**Authors:** Islam M. Saadeldin, Seif Ehab, Jongki Cho

**Affiliations:** 1 Laboratory of Theriogenology, College of Veterinary Medicine, Chungnam National University, 99, Daehak-ro, Daejeon, 34134 Republic of Korea; 2 Research Institute of Veterinary Medicine, Chungnam National University, Daejeon, 34134 Republic of Korea; 3 Biomedical Sciences Program, University of Science and Technology, Zewail City of Science and Technology, Giza, Egypt; 4 Zoology Graduate Program, Department of Zoology, Faculty of Science, Cairo University, Giza, Egypt

**Keywords:** Extracellular vesicles (EVs), Cryo-Electron Microscopy (Cryo-EM), Biomarkers, Multilamellar, Multicompartmental, Vesicular bodies, EVs structures, EVs biogenesis

## Abstract

Extracellular vesicles (EVs) have garnered significant interest in the field of biomedical science due to their potential applications in therapy and diagnosis. These vesicles participate in cell-to-cell communication and carry a diverse range of bioactive cargo molecules, such as nucleic acids, proteins, and lipids. These cargoes play essential roles in various signaling pathways, including paracrine and endocrine signaling. However, our understanding of the morphological and structural features of EVs is still limited. EVs could be unilamellar or multilamellar or even multicompartmental structures. The relative proportions of these EV subtypes in biological fluids have been associated with various human diseases; however, the mechanism remains unclear. Cryo-electron microscopy (cryo-EM) holds great promise in the field of EV characterization due to high resolution properties. Cryo-EM circumvents artifacts caused by fixation or dehydration, allows for the preservation of native conformation, and eliminates the necessity for staining procedures. In this review, we summarize the role of EVs biogenesis and pathways that might have role on their structure, and the role of cryo-EM in characterization of EVs morphology in different biological samples and integrate new knowledge of the alterations of membranous structures of EVs which could be used as biomarkers to human diseases.

## Background

Extracellular vesicles (EVs) have become a focal point in the field of cell biology, as well as in cell-free therapy research, biotechnology, and the pharmaceutical industry. Their potential for clinical applications in diagnostics and therapeutics has generated considerable interest [1, 2]. EVs are heterogeneous nanosized lipid bilayer membranes that are released from different cell types into the extracellular environment. The structural diversity and size of EVs are key determinants in cell signaling, enabling communication between nearby and distant cells. Additionally, EVs act as carriers of bioactive molecules such as nucleic acids, proteins, and lipids, reflecting the characteristics of their originating cells [3–5]. Therefore, EVs have the potential to not only provide insights into physiological states but also serve as valuable tools for understanding pathological functions. This versatility positions them as potential therapeutic cargoes and diagnostic biomarkers for a variety of conditions, including reproductive disorders [6], prenatal genetic diagnosis [7], cancers [8], neurodegenerative diseases [9], and cardiovascular diseases [10]. According to the latest classification by the International Society for Extracellular Vesicles and the updated guidelines for minimal information for studies of extracellular vesicles (MISEV) [11, 12], EVs can be broadly classified into three types based on their size (large vesicles and small vesicles) and mode of biogenesis. These types are as follows: (1) Exosomes (50–200 nm), generated within the endolysosomal system or multivesicular bodies (MVBs); (2) Microvesicles or ectosomes (0.1–1 μm), produced through direct budding from the plasma membrane; and (3) Apoptotic bodies (1–5 μm), arising from dying cells [13, 14]. Despite advances in our understanding of EVs, there are still gaps in our knowledge regarding their structural aspects. EVs have been observed to exhibit a range of structural characteristics, including single, double, or even triple vesicles (multilamellar), as well as other complex structures [15]. Furthermore, multicompartmental vesicles have been identified in biological fluids, and their proportions have been linked to different human diseases. However, the precise mechanisms and functional implications of these structural variations are still poorly understood [16]. The characterization of distinct EV subtypes based on structural aspects is challenging due to the lack of high-resolution techniques. However, cryo-electron microscopy (Cryo-EM) holds great promise for EV characterization, thanks to its exceptional resolution capabilities [17]. In this review, we aim to provide a comprehensive overview of recent studies focusing on the mechanisms of EV biogenesis. Furthermore, we will highlight the potential roles of cryo-EM in characterizing and describing the structure of EVs, with a particular emphasis on the presence of multicompartmental vesicles in various biological samples derived from humans, animals, and microorganisms. Additionally, we will emphasize the significance of evaluating the proportions of these multicompartmental vesicles in biological samples and their correlation with human disease conditions for diagnostic applications.

### Extracellular vesicles (EVs) types and biogenesis

EVs can be categorized into two main classifications based on their size and origin of biogenesis [5]. The first subtype is exosomes, which are formed when the endosomal membrane buds inward to create intraluminal vesicles (ILVs) within endosomes. These ILVs are released as exosomes when endosomes fuse with the plasma membrane. The second subtype is ectosomes, produced by outward budding and fission of the plasma membrane, and the vesicles discharging into the extracellular space. Ectosomes include several vesicle types, such as microvesicles (typically 0.2–1 μm in diameter) and large oncosomes (> 1 μm), as well as during programmed cell death apoptotic bodies release [14, 18, 19]. Additionally, migrasomes released from cancer cells are a subtype of EVs that are still being characterized [20]. The biogenesis of EVs is a complex process involving various machinery responsible for different steps in the formation of exosomes and microvesicles [21]. Exosomes are generated through the intracellular budding of ILVs within the lumen of endosomes during the maturation of multivesicular endosomes (MVEs) in the early endosome process [22]. Golgi apparatus could produce lipid rafts, which facilitate endocytosis and cargoes sorting into ILVs during endosome maturation [23]. The endosomal sorting complex required for transport (ESCRT) machinery, including ESCRT-0, ESCRT-I, ESCRT-II, and ESCRT-III subunits, plays a critical role for the generation of MVEs and ILVs [24–26]. Exosomes and microvesicles both include the ESCRT mechanism [27–29]. It operates through a series of steps, with ESCRT-0 and ESCRT-I subunits clustering ubiquitylated transmembrane cargoes on microdomains of the limiting membrane of MVEs. ESCRT-II and the ESCRT-III subcomplexes then facilitate the budding and fission of these microdomains. HRS, an ESCRT-0 protein involved in early ILV biogenesis, regulates exosome secretion by dendritic cells. It binds to ubiquitylated cargoes and recruits clathrin to the early endosome [30–33]. Thus, the ESCRT machinery components are associated MHC class II proteins have the potential to selectively act on subpopulations of MVEs and ILVs that are involved in exosome secretion [31]. The ESCRT accessory protein ALG-2 interacting protein X (ALIX, also known as programmed cell death 6-interacting protein) and syntenin both influence the ESCRT pathway. The ESCRT-III subunit vacuolar protein sorting-associated protein 32 (VPS32 or CHMP4) and the cargo are linked together by these proteins [34–36]. The ceramide generation by a neutral type II sphingomyelinase is required for the first ESCRT-independent mechanism of exosome biogenesis. This enzyme hydrolyzes sphingomyelin to ceramide, which may do facilitate the generation of membrane subdomains and induce negative curvature of the membranes [37, 38]. The sorting of cargo into exosomal ILVs it’s needed for activation of the Gi-protein-coupled sphingosine 1-phosphate receptor, and this process can be facilitated by the conversion of ceramide to sphingosine 1-phosphate [39, 40]. Additionally, the exosome sorting process is directly assisted by the tetraspanins CD81, CD82, and CD9 [41–46]. Crystal structure analysis found a cone-like structure with an intramembrane lumen that can hold cholesterol has been found that by tetraspanin CD81. This structural characteristic has been reported to be shared by other tetraspanins. The clustering of multiple cone-shaped tetraspanins can lead to the intracellular budding of enriched microdomains, thereby facilitating various stages of exosome generation [47, 48]. Integrins from tetraspanins also play a role in regulating the intracellular routing of cargoes, towards multivesicular endosomes (MVEs), suggesting that dysfunction in tetraspanin function may be various different aspects of exosome formation. Besides tetraspanins, selective targeting soluble or membrane-associated cargo to exosomes are involved in several additional mechanisms. For example, cytosolic proteins can be sequestered into intraluminal vesicles (ILVs) through co-sorting with other proteins, including the chaperones heat shock 70 kDa protein (HSP70) and heat shock cognate 71 kDa protein (HSC70), which are commonly found in exosomes derived from various cell types [49–51]. Microvesicles, on the other hand, are released from the plasma membrane of healthy cells through the rearrangement of lipid components, protein composition, and calcium ion (Ca2+) levels [52, 53]. The asymmetry of membrane phospholipids, particularly the exposure of phosphatidylserine from the inner leaflet to the cell surface, is influenced by Ca2+-dependent enzymes such as aminophospholipid translocases, scramblases, and calpains. These rearrangements result in the physical bending of the membrane and restructuring of the underlying actin cytoskeleton, facilitating membrane budding and microvesicles formation [54–56]. Cholesterol, an abundant lipid component in microvesicles, plays a significant role in their composition. Pharmacological reduction of cholesterol levels has been shown to diminish the ability of activated neutrophils to produce microvesicles [57, 58]. Furthermore, cytoskeletal elements such as the RHO family of small GTPases and RHO-associated protein kinase (ROCK), which regulate actin dynamics, play a crucial role in microvesicle biogenesis in various tumor cell populations [59, 60]. The biogenesis of tumor-derived microvesicles, also known as oncosomes, is closely associated with metabolic changes related to the Warburg effect [37]. In the context of EVs, multicompartmental microvesicles (MCMVs) derived from human umbilical vein endothelial cells (HUVECs) bud from protrusions and encompass vesicular compartments, including multivesicular bodies (MVBs), which can fuse with the MCMV-limiting membrane and release exosomes [61]. Despite these findings, the molecular mechanisms underlying the biogenesis and secretion of EVs are still poorly understood and require further investigation. We suggest that modifications in EVs, including alterations in lipid components and protein composition, may be linked to the structural characteristics of EVs, especially in the case of multicompartmental vesicles, during pathological conditions.

### Cryo-EM is an essential tool for visualizing and analyzing extracellular vesicles (EVs)

One of the most significant challenges in the morphological examination of biological samples is the preservation of reaction intermediates in their metastable states and the achievement of high spatial resolution to elucidate structural and biological functions.

The realm of imaging EVs assumes a profound significance in elucidating the intricate spatial and temporal characteristics inherent to these vesicles. This endeavor contributes to a heightened comprehension of molecular biology, concurrently amplifying our grasp of the prospective diagnostic utilities attributed to these microstructures. In the context of in vitro EV imaging, researchers are afforded an avenue to delve into the tangible attributes of EVs, encompassing the dynamics of their release and uptake mechanisms, as well as the identification of surface-bound biomarkers [62]. Over time, a multitude of imaging modalities and labeling techniques have emerged, empowering researchers to monitor EVs both in vitro and in vivo conditions [63, 64]. These techniques encompass a repertoire of methodologies, notably scanning electron microscopy (SEM), transmission electron microscopy (TEM), cryogenic electron microscopy (Cryo-EM), and atomic force microscopy (AFM) [65, 66].

**Fig. 1:**
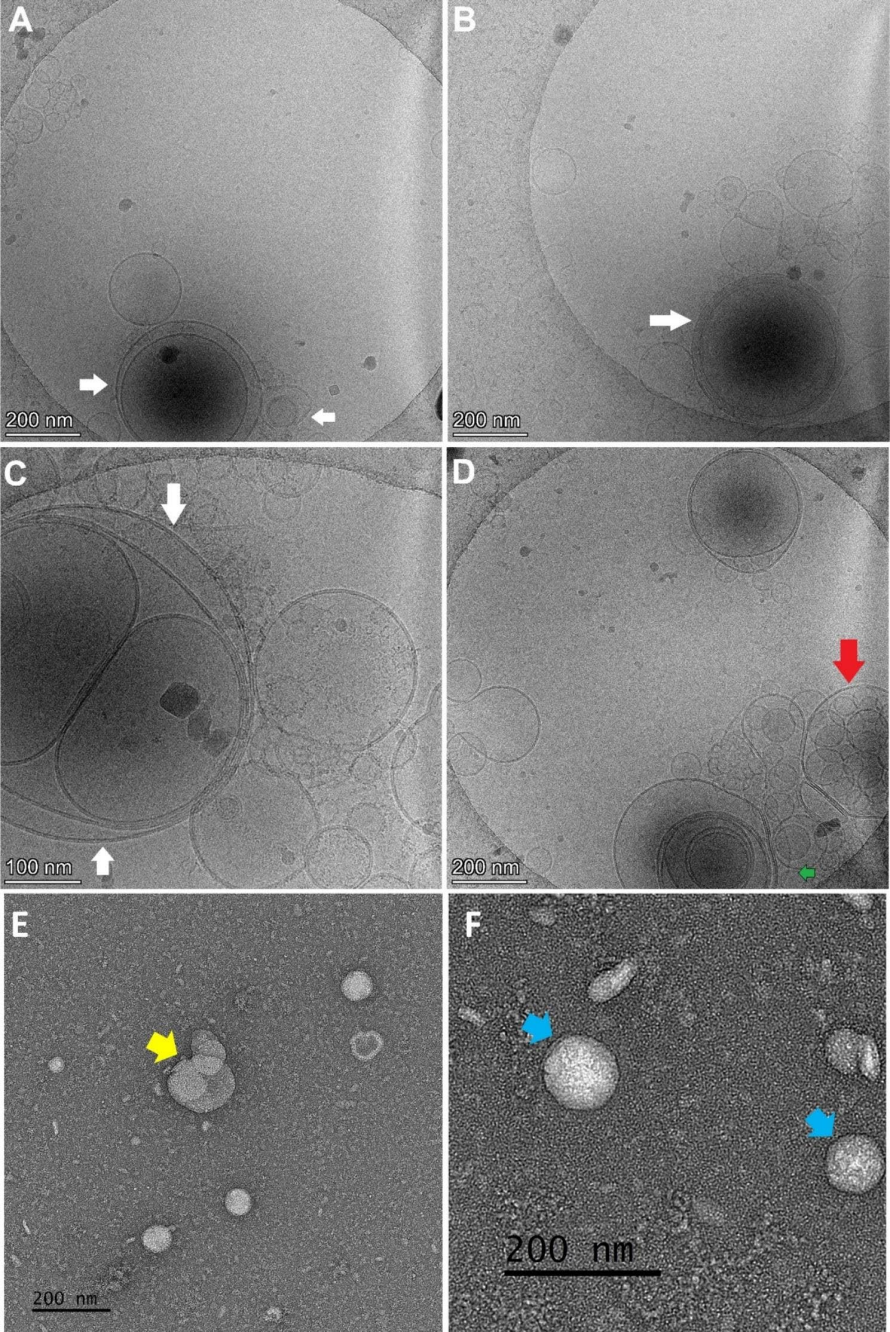
Comparison between the image quality of cryo-TEM and conventional TEM in the same biological sample (egg yolk). Cryo-TEM reveals distinct types of extracellular vesicles (EVs). (**A**) Unilamellar vesicles, (**B**) Bilamellar vesicles with white arrows indicating the presence of multilamellar structures. (**C**) Multicompartmental vitellovesicles depicted by red arrows, where more than ten vesicles are packed inside a larger vesicle. (**D**) A multilamellar vesicle denoted by a green arrow. The scale bar is 200 nm for all panels except panel C is 100 nm. (**E**) Conventional TEM images show low resolution multicompartmental vesicle (yellow arrow), and (**F**) bicompartmental vesicles (blue arrows). It is well noted that the images quality are different and more advantages are towards the use of cryo-EM. These images have not been previously published

Cryo-EM has emerged as a powerful technique for studying EVs, revealing diverse structures, including multiple lipid bilayers, with exceptional detail. Moreover, cryo-EM can differentiate between vesicles and debris as well as probe vesicle morphology and surface structure [63]. Additionally, cryo-TEM proves particularly valuable for imaging either extremely small or notably large collections of vesicles, especially those situated close to the detectable limits of NTA instruments. This technique enables the visualization of surface characteristics, rendering it the preferred approach for discerning variations within vesicle subgroups. Moreover, cryo-TEM demonstrates its utility when investigating samples contaminated by additional non-vesicular elements, which might emerge during the isolation procedure [63]. Cryo-EM has been extensively employed in various studies to characterize and describe the structures and morphological features of EVs in different biological samples (Tables 1 and 2). This technique enables the visualization of a wide range of EVs with varying sizes and diverse morphologies. It also provides insights into external structures embedded in vesicle membranes and the encapsulation and internalization of cargo within EVs. Cryo-EM has uncovered different EV structures, such as single vesicles, double vesicles, triple vesicles, double-membrane vesicles, vesicles with electron-dense cargo in the lumen, joint vesicles, vesicles with broken membranes, short tubules, and long tubules [14] (Fig. 1A-D). Cryo-EM stands out as the optimal method for EV research due to several advantages. First, it avoids the use of chemical fixatives such as dehydration and heavy metals, preserving the samples in their natural hydrated state. Second, it allows for the observation of EVs at near-native size and ultrastructure. Third, it offers rapid imaging for experienced operators and achieves high resolution. Fourth, when combined with immuno-gold labeling, cryo-EM provides additional information and detects morphological details of EVs, surpassing the capabilities of conventional techniques. In particular, when combined with single-particle analysis, cryo-EM can achieve atomic and near-atomic resolution, further enhancing its analytical power [17]. Furthermore, cryo-EM is well-suited for capturing images of EVs with membranous structures and lumens, facilitating the precise localization of specific proteins, which is crucial for studying the biological functions of EVs [67]. Cryo-transmission electron microscopy (cryo-TEM) is an additional valuable technique that provides a near-native view of exosomes, enabling analysis of their internal structure. This method involves block preparation, thin sectioning, and electron tomography. Furthermore, an automated serial sectioning technique utilizing a focused ion beam with a thickness of 15 nm has been employed to observe the three-dimensional structure of exosomes of various sizes [68]. Furthermore, cryo-EM has revealed the morphological plasticity of EVs, demonstrating their ability to change shape and/or move when isolated from human mast cell (HMC-1) cultures and human ejaculate samples [69]. Therefore, cryo-EM is the preferred choice for imaging EVs and analyzing their morphological aspects. Its ability to capture the heterogeneity of vesicles, provide high-resolution details, and preserve their natural state makes it an indispensable tool for EV research. Unlike the spherical detailed morphology of EVs revealed by Cryo-EM, EVs are visualized with conventional TEM appear as double layered cup-shaped membrane structure with their diameter ranges between 30 and 100 nm with unclear internal contents details [70] (Fig. 1E-F). Moreover, the quality of EVs TEM imaging is operator and protocol dependent, which further limits it use for detailed description of ultrastructure of EVs [71]. Other methods such as SEM and AFM are restricted to EVs surface analysis and used for diameter analysis or topography [72] (Table 3). Collectively, these comparison makes the cryo-EM is the method of choice when categorizing EVs according to the internal structures.

**Table 1:** Studies on structural aspects and proportions of multicompartmental vesicles derived from EVs highlighting their potential correlation with human diseases and relevance for clinical diagnostics

Human samples	Examined particles	Unilamellar	Bi- and trilamellar	Multicompartmental	Condition	Ref.
Cerebrospinal fluid	75	54.4%	8.1%	36.5%*	Parkinson’s Disease (PD)	[76]
Cerebrospinal fluid	85	82.3%	9.4%	8.3%	Other neurological diseases	[76]
Cerebrospinal Fluid		40%	60%	NA	Traumatic brain injury	[91]
Ejaculates	1110	76.9%	19.6%	3.5%	Normal conditions	[77]
Mast cell	1724#	81.7%	1.1%	1.3%	Conditioned medium	[80]
Myelinosome-like vesicles in seminal plasma		59%	20–30%	< 1%	Normal conditions	[88]
Plasma\total Blood §		15%\20%	70%\81%	5%\10%	Diagnostic/prognostic breast cancer	[92]
Visceral adipose tissue (VAT) \ subcutaneous adipose tissue (SAT)‡		65.40%\84.70%	22.40% \8.10%	8.60% \4.20%	Obesity and type 2 diabetes mellitus	[130]

*: Percentage of multicompartmental vesicles of PD are statistically higher than other CSF samples at P < 0,05 (Data were analyzed with Mann–Whitney test) [76]

#: the authors considered more categories including the oval, small tubules, incomplete, or pleomorphic vesicles with a proportion of around 16%

§: the percentage for Plasma and total Blood samples from breast cancer patients

‡: The percentage numbers for VAT and SAT with obesity and type 2 diabetes

**Table 2:** Recent studies utilizing Cryo-EM to isolate and characterize extracellular vesicles (EVs) and investigate their structural aspects, including the presence of multicompartmental vesicles, in various biological samples from humans, animals, and microorganisms

Sample source	Species	Conditions	Ref.
Human leukemia monocytic cell line (THP-1)	Human	Lipopolysaccharide (LPS) and starvation conditions	[89]
Follicular Fluid	Human	Normal fertile	[131]
Plasma, MDA231, HVT, THP1 cell lines	Human	Diagnostic tools	[93]
Plasma	Human	Dementia with Lewy bodies disease	[94]
Plasma	Human	Clinical applications	[95]
Plasma	Human	Gaucher disease	[132]
Semen	Human	Normal condition	[78]
Seminal plasma	Human	Normal condition	[132]
3T3-L1 cells adipocytes	Human	Hypoxic conditions	[133]
Umbilical cord mesenchymal stem cells	Human	Normal conditions	[96]
Human umbilical vein endothelial cells (HUVECs)	Human	Normal conditions	[61]
Coronin 1 C-null cell line	Mouse	Melanoma metastasis	[99]
Blood sample	Mouse	Atherosclerosis Disease	[97]
Superior cervical ganglion	Mouse	Sympathetic cultures	[98]
Synovial fluid	Equine	Isolation of CD44	[129]
Egg yolk	Chicken	Embryotrophic effect on mammalian embryos.	[74]
Marine toxic dinoflagellate *Alexandrium minutum*	Microorganism	Axenic culture	[100]
Dictyostelium cells, Ax-2 strain	Microorganism	Vitreous ice environment	[101]

### Multicompartmental vesicles in health and diseases

Recently, Broad et al. [73] highlighted the scarcity of research regarding the biogenesis and roles of multilamellar extracellular vesicles (EVs) reported in several recent studies. Our findings further support the need for additional research on the mechanisms underlying the formation of multilayered vesicles. Our recent observations have revealed the presence not only of unilamellar or multilamellar vesicles but also of multicompartmental and multivesicular vesicles [74]. Multicompartmental vesicles are characterized by the presence of smaller internal vesicles arranged non-concentrically. As depicted in Fig. 1, these vesicles can exhibit unilamellar, bilamellar, or multilamellar structures. Interestingly, vesicles measuring approximately 400 nm in size can contain various compartments consisting of smaller unilamellar or bilamellar vesicles. Additionally, we have observed intraluminal vesicles resembling multivesicular bodies (MVBs), with each vesicle housing numerous small intraluminal vesicles (< 100 nm) [75]. In our study, out of the 2415 examined vesicles, the majority were unilamellar vesicles (93%), followed by multicompartmental vesicles (4.5%), bilamellar vesicles (2%), and multilamellar vesicles (0.5%). The presence of multicompartmental vesicles has been reported in various biological samples, including the cerebrospinal fluid of patients with Parkinson’s disease [76], human ejaculated semen [77], prostate secretions [78], THP-1 cells [79], and mast cells [80] conditioned media [49]. The elevated proportion of multicompartmental vesicles in Parkinson’s disease (PD) compared to other neurological disorders (36.5% vs. 8.3%) as indicated in Table 1 raises intriguing questions about the potential utility of multicompartmental vesicles as a diagnostic marker for PD or their involvement in aberrant EVs biogenesis in this condition.

**Table 3:** A comparison of available microscopy techniques to visualize the EVs

Method	Resolution / detection limit	Physical basis	Environment	EVs morphology	Detailed internal structure	Fine membrane structure
SEM	1 nm	Emission of electrons	Vacuum	Spherical but agglomeration can be occurred due to the drying process	No	No
AFM	1nm^a^ (XY), 0.1 nm^a^ (Z)	Physical interaction with sample	Vacuum, air, and liquid	Globular	No	No
TEM	0.1 nm	Scattering of electrons	Vacuum	Rounded or cup-shaped	No	Low quality
Cryo-EM	< 15Å	Scattering of electrons in low temperature	Vacuum	Rounded and 3D structure can be composed	Yes	High quality

SEM: Scanning electron microscopy. AFM: Atomic force microscopy. TEM: Transmission electron microscopy. Cryo-TEM: Cryogenic transmission electron microscopy

### The proposed mechanisms of multilamellar and multicompartmental vesicles formation

The formation of multicompartmental vesicles could be due to the following reasons:

#### Biogenesis and physiological alterations

The fundamental structure of the membrane is a phospholipid bilayer, which forms a stable barrier between the two aqueous compartments. Therefore, the lipid composition has been shown to influence the physical phase of lipids in cellular membranes in various diseases [81]. It has been demonstrated that dysregulation of lipid homeostasis and altered distribution in liver fibrosis can be revealed through multimodal nonlinear optical microscopy [82]. Moreover, changes in the phospholipid fatty acid composition of membranes lead to modified membrane fluidity and cellular signaling. Increased levels of long-chain polyunsaturated fatty acids (LC-PUFAs) are thought to enhance membrane fluidity [83]. Furthermore, there exists a correlation between the membrane bilayer and the circadian clock, which holds significant implications for circadian rhythm disorders and related chronic diseases like obesity, diabetes, and cardiovascular disease [84].

Communication through vesicles and cargo transfer is crucial for all physiological processes, with an illustrative example being the central nervous system’s utilization of synaptic and dendritic vesicles for signal transmission. Membrane dysfunction is a prevalent mechanism that contributes to neuronal vulnerability in sphingolipid storage disorders associated with neurological impairment [85]. Additionally, neurons encounter specific difficulties in effectively managing the supply and retrieval of the plasma membrane (PM) in their distal regions, a process known as PM turnover. Dysregulation of this process may lead to dendritic pathology observed in various neurodegenerative diseases [86]. Genetic diseases related to ether lipid (mutations in GNPAT, FAR1, or AGPS) and sphingolipid synthesis (mutations in SPTLC1 or SPTLC2) have highlighted the importance of small differences in lipid chemical structures. Ether lipids, which differ only in their sn-1 fatty acid linkages, play a crucial role, and deficiency in these lipids leads to severe diseases [87].

Most features of EVs not only reflect the physiological state but also indicate their pathological functions. The structure and packaging of EVs play a crucial role, as they contain bioactive cargo within multiple layers of the membrane, offering protection against degradation in the extracellular space. Multicompartmental vesicles, in particular, enable the vesicle contents to evade lysosomal degradation in the recipient cytoplasm or facilitate their transport to the nucleus [61]. When studying EVs isolated from myelinosomes of TM4 Sertoli cells and human seminal plasma samples, cryo-EM analysis revealed diverse morphological aspects and proportions of multicompartmental vesicles. In both samples, the distribution of vesicle types was as follows: unilamellar (59%), bi- and trilamellar (20–30%), and multicompartmental (< 1%) [88]. Similarly, cryo-EM analysis of human ejaculate EVs revealed a wide range of morphologies, identifying 11 subcategories of membrane structures. Among these, 59% were single vesicles, while the remaining 41% comprised more complex assemblies, including various membrane compartments [77]. Notably, human seminal fluid contains small exosome-like vesicles called proteasomes, which exhibit morphological diversity. Multiple vesicles often contain secondary and occasionally tertiary vesicles of smaller size, while some vesicles appear as singular entities. The majority of vesicles exhibit a nearly round shape, with some being completely round or egg-shaped [78]. In another study, EVs isolated from the human leukemia monocytic cell line THP-1 under lipopolysaccharide (LPS) and starvation conditions displayed heterogeneity in terms of structure and size. Cryo-EM analysis revealed the presence of single and double-layered vesicles, as well as multivesicular bodies containing smaller vesicles [89]. Moreover, cryo-EM imaging of EVs derived from brain tissue revealed the presence of multi-lamellar enclosures in small EVs [90]. Cryo-EM was also utilized to investigate EVs isolated from human cerebrospinal fluid (CSF). Among the 20 individual EVs analyzed, 60% exhibited multi-membrane structures, while the remaining 40% had single membranes [91]. Plasma EVs isolated from breast cancer patients exhibited a range of morphologies, including single vesicles, double vesicles, multilayer vesicles, double-membrane vesicles, and vesicles with electron-dense cargo in the lumen. The prevalence of single vesicles decreased from 91 to 37% in total blood vesicles, while the proportion of double vesicles increased from 3 to 20% and double-membrane vesicles increased from 0 to 22% in patients with breast cancer. [92]. Further diversity in EV morphology was observed in follicular fluid samples using cryo-EM. The analysis revealed the presence of single vesicles (55%), double vesicles (13%), triple vesicles (6%), oval vesicles (13%), vesicle sacs (2%), pleomorphic membrane structures (5%), lamellar bodies (3%), small tubules (1%), and large tubules (1%). These results were obtained through high-speed centrifugation at 100,000 × g without differential steps [64]. Under hypoxic conditions, adipocytes (3T3-L1 cells) displayed the presence of multicompartmental vesicles compared to normoxic conditions [65]. Additionally, EVs isolated from the human mast cell line HMC-1 have shown diverse EV morphology and structures, categorized into nine different groups based on their size and shape (single vesicles 81.7%, double vesicles, triple vesicles, small double vesicles, oval vesicles, incomplete vesicles, small and large tubules, and pleomorphic vesicles). Three additional morphological features were also found in exosomes regardless of their morphological classification (coated vesicles 3.7%, filamentous vesicles 0.5%, and electron-dense vesicles) [80]. Cryo-EM images of circulating microparticles (MPs) withdrawn from the blood of a healthy subject and MDA231, HVT, and THP1 cell lines show smaller vesicles encapsulated inside larger MPs, creating multi-layered MPs [93]. Plasma EVs from patients with dementia and Lewy bodies have been identified using cryo-TEM multicompartmental vesicles [94]. In addition, human plasma EVs isolated by protein–organic solvent precipitation showed multilayered EVs and multicompartmental vesicles [95]. Furthermore, cryo-EM analysis of EVs derived from umbilical cord mesenchymal stem cells (UCMSCs) under normal conditions confirmed the presence of pooled EVs fractions [96]. However, the lymph from atherosclerotic mice exhibited a higher concentration of EVs. Cryo-EM imaging, without annexin V gold nanoparticles, revealed the absence of phosphatidylserine (PS) on the outer membrane surface and the presence of multicompartmental vesicles [97]. In a mouse superior cervical ganglion model, EVs derived from sympathetic cultures were isolated and characterized using cryo-EM. This analysis revealed distinct EVs morphologies and the presence of multicompartmental vesicles, also referred to as “EVs inside another EVs” [98]. Moreover, EVs derived from a Coronin 1 C null cell line originating from a brain metastasis in a male PBT-1 C mouse displayed multicompartmental EVs, as observed through TEM [99]. Cryo-TEM analysis of EVs isolated from equine synovial fluid demonstrated the presence of EVs with a collective morphology under different centrifugation conditions. The proportions were found to be 4.41% at 10,000 g and 1.27% at 100/200,000 g [74]. EVs isolated from microorganisms, specifically the toxic dinoflagellate *Alexandrium minutum*, displayed distinct morphological groups when observed using cryo-TEM. These groups included rounded, rounded electron-dense, lumen electron-dense, double, and irregular EVs, with an average diameter of 0.36 μm [100]. Furthermore, EVs isolated from Dictyostelium cells (Ax-2 strain) and analyzed using cryo-EM exhibited specific configurations that remained conserved in the vitreous ice environment during growth. These configurations included broken vesicles, large EVs inserted inside larger vesicles, small EVs inside multivesicular body-like vesicles, and EVs prone to fusion) [101].

Interestingly, several reports have shown that double-membrane vesicles (DMVs) are associated with positive-sense ssRNA (+ RNA) virus infections in eukaryotic cells due to the hijacking of secretory pathways and the induction of membrane remodelling [102]. The ectopic expression of certain non-structural viral proteins, such as transmembrane or membrane-tethering domains, or viral RNAs has been suggested to play crucial roles in DMV biogenesis [103, 104].

**Fig. 2:**
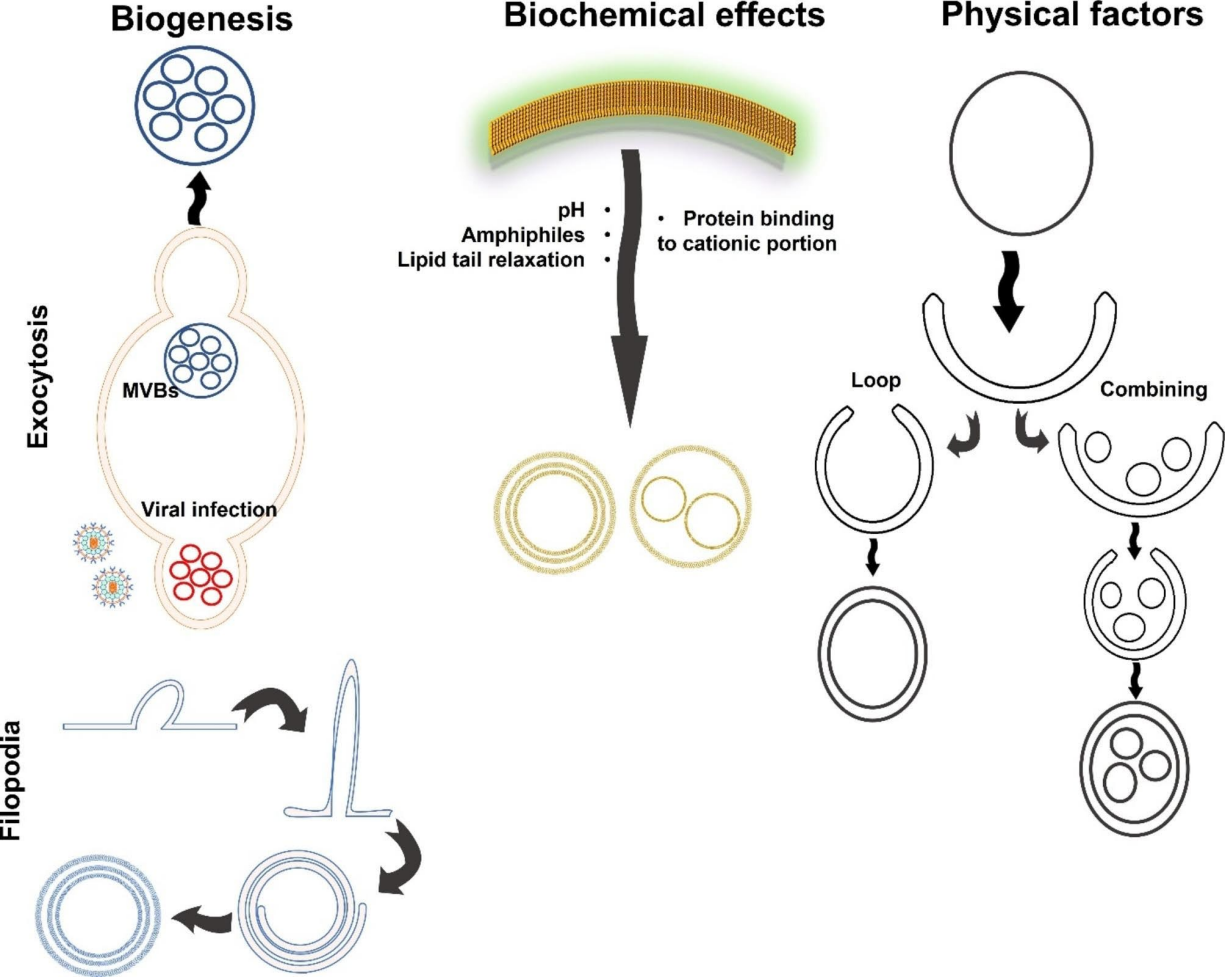
A proposed diagram of multicompartmental EVs formation. Multilamellar and multicompartmental vesicles can be caused during the biogenesis where an altered exocytosis such as viral infection or rolling and folding of the elongated filopodia. They can also be formed due to biochemical changes and physical pressure caused by the ultracentrifugation forces of less stable big-sized vesicles

Similarly, the folding and curvature of the long cisternae or filopodia can result in the formation of multi-layered or multicompartmental vesicles (Fig. 2).

#### Biochemical effects

Unilamellar vesicles can be artificially transformed from their initial unilamellar state into multilamellar vesicles through the reduction of membrane rigidity using nonionic surfactant-like polymers, such as n-alkyl-ethylene oxide polymers [105]. Moreover, protein binding (e.g., epidermal growth factor (EGF) to the surface of cationic unilamellar lipid vesicles, can trigger a layer-by-layer self-assembly process, resulting in the conversion of unilamellar vesicles into multilamellar vesicles [106]. Additionally, the use of bilayer-forming amphiphiles dissolved in water-miscible organic solvents allows for the trapping of smaller vesicles by unilamellar vesicles or liposomes, leading to the formation of multicompartmental vesicles [107–109]. Importantly, changes in the pH of the vesicle environment can impact the ionic properties of the bilipid layers, potentially leading to the transformation of vesicles into multilamellar or multicompartmental structures [110]. Interestingly, multivesicular vesicles (MVVs) are non-concentrically arranged vesicles and can be formed through different biochemical methods [107]. The molecular-recognition processes mediated by lipophilic receptors and a substrate such as biotin-streptavidin complex can be used to produce a multicompartmental aggregate of tethered vesicles encapsulated within a large bilayer vesicle [111]. These encapsulated aggregates are called vesosomes. Encapsulation is achieved by unrolling bilayers from cochleate cylinders which are tethered to the aggregate by biotin-streptavidin coupling [111, 112]. Also, the formation of an aqueous dispersion of MVVs based on the transformation of rigid, interdigitated sheets into spherical vesicles with a high Tm (DPPC with Tm ≈ 41 °C, or DSPC (1,2-distearoyl-sn-glycero-3-phosphocholine) with Tm ≈ 55 °C)9 in the presence of a high concentration of ethanol (≈ 3 M) if the sample is kept at T < Tm [113]. In addition to the formation of individual MVVs can be controlled by hydration of a dry film of bilayer-forming amphiphiles deposited on a solid surface [114]. Furthermore, another method requires the initial preparation of a water-in-oil-in-water (w/o/w) double emulsion. This type of emulsion consists of dispersed micrometer-sized, amphiphile monolayer-coated aqueous droplets inside oil droplets that are dispersed in a bulk aqueous solution (the oil droplets also being coated by a monolayer of amphiphiles used for the formation of a dispersion of heterogeneous MVVs from an initial w1/o/w2 double emulsion [115]. However, the use of w1/o/w2 droplets prepared inside a microfluidic channel of a specifically designed chip used for the formation of MVVs [116] The method of transfer of water droplets from a w/o emulsion into an aqueous solution used for the formation of a dispersion of MVVs [117] The formation of a dispersion of MVVs by adding a solution of bilayer-forming amphiphiles in a water-miscible organic solvent to an aqueous dispersion of separately prepared vesicles [108]. The formation of MVVs also can done by sequential microfluidic jet blowing method on planar bilayers of amphiphiles [118] The formation of giant MVVs through complementary vesicle binding and subsequent vesicle internalization by amphiphiles with a bulky or charged polar head group are embedded in the vesicle membranes to increase the colloidal stability of vesicle dispersions [119].

#### Physical impacts

We also suggest that the ultracentrifugation forces and continuing pressure may be able to squeeze some of the less rigid or flaccid vesicles and remodulate the morphology of the vesicles into multi-layered [120] or internalize small vesicles into multicompartmental ones [121–123] as shown in Fig. 2. In addition, formation of MVVs can be achieved through mixing of two vesicle dispersions, one prepared from high Tm bilayer-forming amphiphiles, the other prepared from low Tm amphiphiles [124]. Light irradiation and heating can prompt the vesicle formation. Heat can induce formation of single giant unilamellar vesicles (GUV) by directed infrared (IR) laser heating [125]. Furthermore, GUV can be formed through localizing IR heating of spin-coated lipid films. The GUV are formed due to interaction between the charged and neutral lipid species, as well as from a complex lipid mixture, in various ionic strength conditions [126]. Micrometer-size vesicle formation can be triggered by UV light through assembling a mixture of particles consisting of crumpled phospholipid multilayer membranes involving a photoactive amphiphilic compound composed of 1,4-bis(4-phenylethynyl)benzene (BPEB) units [127]. In addition, electric fields can be used to promote conversion of lipid molecular self-assemblies into GUVs through using nonhomogeneous electric field generated by point-to-plane electrodes [128].

### Perspectives and conclusion

Extracellular vesicles (EVs) have emerged as promising entities in biomedical science due to their potential diagnostic and therapeutic applications. Previous studies have recognized the significance of the bioactive molecules carried by EVs. However, there remains a need for extensive research into the structural and morphological aspects of EVs and their association with human diseases. Standardized criteria and terminology for EVs morphology are lacking, particularly in differentiating multicompartmental EVs from multilamellar EVs. Various terms have been used to describe multicompartmental EVs, including MVB containing smaller vesicles (possibly exosomes) [89] or multi-lamellar enclosed in small vesicles [97], EVs inside other EVs [98], or vesicles encapsulated inside bigger MPs, creating multi layered MPs [93] EVs collections [129] pooled EVs fractions [96]. Despite the differences in terminology, these terms refer to the same multicompartmental EVs.

Given these discoveries, it is of utmost importance for researchers studying EVs to report the quantitative variances or proportions of these abnormal bilipid multicompartmental and multilayered vesicles in both healthy and diseased states, and to share this data with the scientific community. Furthermore, the visualization of multicompartmental vesicles larger than 500 nm would be beneficial in differentiating migrasomes from large ectosomes [21]. Investigating the morphology and distribution of these multicompartmental EVs is crucial for comprehending their mechanisms of biogenesis, functions, and potential associations with specific disease conditions.

This review highlights the inadequate research conducted on the origins and functions of multicompartmental EVs, which have been observed in human biofluids, biological samples, as well as in animal and microorganism samples, across diverse physiological and pathological contexts. There is a clear need for further studies aimed at unraveling the mechanisms underlying the biogenesis, biological roles, and importance of multicompartmental EVs. Such investigations hold the potential to reveal novel opportunities for therapeutic strategies, identify therapeutic targets, and develop disease biomarkers.

## Data Availability

The proteomics data of the egg yolk nanovesicles are available in the ProteomeXchange dataset PXD038679 [doi:10.25345/C5RV0D524]. The RNA sequencing dataset is available in the NCBI GEO database (https://www.ncbi.nlm.nih.gov/geo/query/acc.cgi?acc=GSE219218).

## List of abbreviations

BPEB: 1,4-bis(4-phenylethynyl)benzene
Cryo-EM: cryogenic electron microscopy
CSF: Cerebrospinal fluid
DPPC: 1,2-dipalmitoyl-sn-glycero-3-phosphocholine
DSPC: 1,2-stearoyl-sn-glycero-3-phosphocholine
ESCRT: endosomal sorting complex required for transport
EVs: extracellular vesicles
GUV: giant unilamellar vesicles (GUV)
ILVs: intraluminal vesicles
MEVs: multivesicular endosomes
MVBs: multivesicular bodies
MVVs: multivesicular vesicles
PD: Parkinson’s disease
TEM: transmission electron microscopy
Tm: sold main phase transition temperature
UV: Ultraviolet
